# Vegetation restoration restructures soil sulfur allocation and sulfur-cycling functional potential in the Mu Us Sandy Land

**DOI:** 10.3389/fmicb.2026.1845938

**Published:** 2026-06-04

**Authors:** Xuetong Wang, Ning Yang, Yueyue Hu, Xuerong Wang, Yang Wu, Aiping Zhang, Yanan Wang

**Affiliations:** 1 College of Natural Resources and Environment, Northwest A&F University, Yangling, China; 2 College of Environmental Sciences, Sichuan Agricultural University, Chengdu, China; 3 School of Life Science, Shanxi Normal University, Taiyuan, China; 4 Advanced Institute of Natural Sciences, Beijing Normal University, Zhuhai, China; 5 School of Environment, Beijing Normal University, Beijing, China; 6 Institute of Environment and Sustainable Development in Agriculture, Chinese Academy of Agricultural Sciences, Beijing, China

**Keywords:** metagenomics, microbial community, sulfur pool allocation, sulfur-cycling functional genes, vegetation restoration

## Abstract

Vegetation restoration in semi-arid sandy ecosystems can alter soil sulfur cycling not only through changes in sulfur stocks, but also through shifts in the partitioning between organic sulfur and sulfate and their microbial regulation. Here, we investigated soil sulfur pool allocation and sulfur-cycling functional potential along a five-stage vegetation restoration gradient in the Mu Us Sandy Land by integrating sulfur fraction measurements with metagenomic analyses. Vegetation restoration markedly reshaped the soil physicochemical and microbial context, as reflected by lower pH and higher TN, microbial biomass carbon, and enzyme activity in restored soils. In contrast, sulfur pools responded asynchronously: total sulfur and organic sulfur declined substantially from bare sandy land to restored vegetation types, whereas sulfate showed a weaker and comparatively more stable response. At the functional level, dominant sulfur-cycling genes were generally more abundant in bare sandy land, declined across restored vegetation types, and showed only partial recovery in forestland, indicating that restoration reorganized sulfur-cycling functional composition rather than uniformly enhancing sulfur-cycling potential. Taxonomically, dominant sulfur-cycling genes were consistently affiliated mainly with *Actinomycetota* and *Pseudomonadota*, but restored vegetation types exhibited more partitioned host compositions, with greater contributions from *Acidobacteriota, Chloroflexota*, and, for some genes, *Thermoproteota*. MAG-based analyses further showed that key sulfur-cycling genes were phylogenetically widespread but unevenly distributed across specific host lineages. Co-variation and Mantel analyses showed that sulfur-cycling genes formed coordinated functional modules and were most strongly associated with soil sulfur pools and fractions. Overall, vegetation restoration in the Mu Us Sandy Land primarily reshaped sulfur allocation and sulfur-cycling functional potential rather than promoting simple sulfur accumulation. These findings highlight that sulfur recovery in sandy drylands is better characterized by pool reallocation and functional reorganization.

## Introduction

Dryland ecosystems occupy a substantial proportion of the global terrestrial surface and are projected to face increasing aridity and stronger hydroclimatic constraints under ongoing climate change, with profound consequences for vegetation recovery and ecosystem biogeochemical functioning (Huang et al., 2017; Maestre et al., 2016). In these water-limited systems, the success of ecological restoration is no longer evaluated solely by aboveground vegetation recovery, but increasingly by the re-establishment of belowground nutrient cycling processes, which underpin long-term ecosystem productivity and stability (Costantini et al., 2016; Kimmell et al., 2023; Young et al., 2021). Compared with carbon (C), nitrogen (N), and phosphorus (P), sulfur (S) has received remarkably limited attention in restoration studies, despite its essential roles in plant protein synthesis, enzymatic function, and microbial metabolism (Kertesz and Mirleau, 2004; Wilhelm Scherer, 2009). Moreover, declining atmospheric sulfur deposition, together with increasing nutrient demand associated with biomass accumulation during vegetation restoration, may increase the risk of sulfur limitation in nutrient-poor sandy soils and semi-arid landscapes (Aas et al., 2019; Cheng et al., 2022).

A central challenge in evaluating soil sulfur recovery is that total sulfur (TS) does not reliably reflect biologically available sulfur. In most soils, sulfur is predominantly stored as organic sulfur (OS), whereas sulfate sulfur (${\text{SO}}_{4}^{2}$-S) constitutes the directly plant-available and more mobile fraction (Narayan et al., 2023; Wilhelm Scherer, 2009). These two pools are dynamically interconnected through microbial mineralization, immobilization, and enzymatic transformations. Consequently, restoration-induced changes in organic matter inputs, rhizosphere activity, and soil environmental conditions may alter the partitioning between OS and ${\text{SO}}_{4}^{2}$-S without being synchronously reflected in TS. As a result, reliance on TS alone may obscure the actual effects of restoration on sulfur availability and may lead to a “TS-availability decoupling” pattern along restoration gradients (Ranadev et al., 2023). Therefore, an explicit pool-resolved framework that partitions TS into ${\text{SO}}_{4}^{2}$-S and OS is needed to distinguish sulfur storage from sulfur availability in soils.

Microorganisms provide the mechanistic link between sulfur pool partitioning and ecosystem functioning because they directly regulate the transformation between storage and available sulfur pools (Kertesz and Mirleau, 2004; Zhou et al., 2025). In soils, this regulation is mainly mediated through three processes: dissimilatory sulfate reduction, sulfur oxidation, and organic sulfur mineralization. Dissimilatory sulfate reduction represents the reductive branch of the sulfur cycle and is commonly inferred from *dsr*-related genes (Müller et al., 2015; Muyzer and Stams, 2008; Pester et al., 2012), whereas sulfur oxidation is primarily associated with the *Sox* pathway, making *sox*-related genes informative indicators of oxidative potential (Friedrich et al., 2005; Meyer et al., 2007; Moreau et al., 2010; Petri et al., 2001; Tourna et al., 2014). Organic sulfur mineralization is more directly linked to sulfur availability because it continuously releases sulfate through the decomposition of sulfate esters and sulfonates (Kertesz, 2000; Wilhelm Scherer, 2009). For example, *ssuD*, which encodes alkanesulfonate monooxygenase, is involved in organosulfonate utilization and responds to sulfate limitation, making it a useful marker for tracking sulfur acquisition from sulfonates (Park et al., 2020). Importantly, organic sulfur should not be viewed as a static storage pool; rather, under suitable environmental conditions, it can actively participate in internal soil sulfur cycling and regulate sulfate supply (Borodina et al., 2000; Kertesz, 2000). Taken together, resolving microbial sulfur-cycling functional potential provides a key mechanistic basis for understanding sulfur pool reallocation and changes in sulfur availability during vegetation restoration.

In sandy dryland ecosystems, vegetation restoration can alter soil organic carbon accumulation, moisture status, pH, salinity/ionic environment, and microbial attributes, and these changes collectively define the environmental context governing sulfur pool partitioning and sulfur-cycling functional potential (Kimmell et al., 2023; Maestre et al., 2016; Muñoz-Rojas, 2018). These restoration-induced changes can further reorganize rhizosphere carbon inputs and oxygen diffusion, creating spatially heterogeneous microsites where sulfate reduction, sulfur oxidation, and organic sulfur transformation coexist and interact, with important consequences for internal sulfur cycling and sulfur availability; however, plant-available sulfate may still remain constrained when microbial immobilization or organic sulfur accumulation predominates (Friedrich et al., 2005; Muyzer and Stams, 2008; Pester et al., 2012). Nevertheless, restoration studies remain fragmented, often focusing either on soil physicochemical properties and sulfur fractions or on functional genes alone, without integrating sulfur pool partitioning, microbial functional potential, and key environmental drivers along the same restoration gradient (Costantini et al., 2016; Muñoz-Rojas, 2018; Young et al., 2021). This gap is particularly important in semi-arid sandy ecosystems, where low organic matter content, together with high sulfate mobility and leaching risk, makes soil sulfur availability highly sensitive to subtle shifts in organic sulfur turnover, microbial acquisition strategies, and environmental filtering (Wilhelm Scherer, 2009).

Here, we investigated the mechanisms underlying soil sulfur pool reallocation and microbial sulfur-cycling functional potential along a vegetation restoration gradient in the Mu Us Sandy Land. We quantified soil sulfur fractions (TS, ${\text{SO}}_{4}^{2}$-S, and OS), characterized sulfur-cycling functional modules and key microbial groups using metagenomic data, and further identified the dominant environmental drivers of these changes. Specifically, we aimed to address the following questions: (1) How does vegetation restoration influence soil sulfur pool partitioning, particularly the redistribution between OS and ${\text{SO}}_{4}^{2}$-S? (2) How do sulfur-cycling functional potential and key microbial groups vary along the restoration gradient? (3) Which environmental factors dominate sulfur pool reallocation and microbial functional restructuring during vegetation restoration? Answering these questions will help clarify the mechanisms of soil sulfur recovery during dryland restoration and provide a theoretical basis for evaluating nutrient recovery in arid and semi-arid ecosystems.

## Materials and methods

### Study area and sample collection

**Figure 1:**
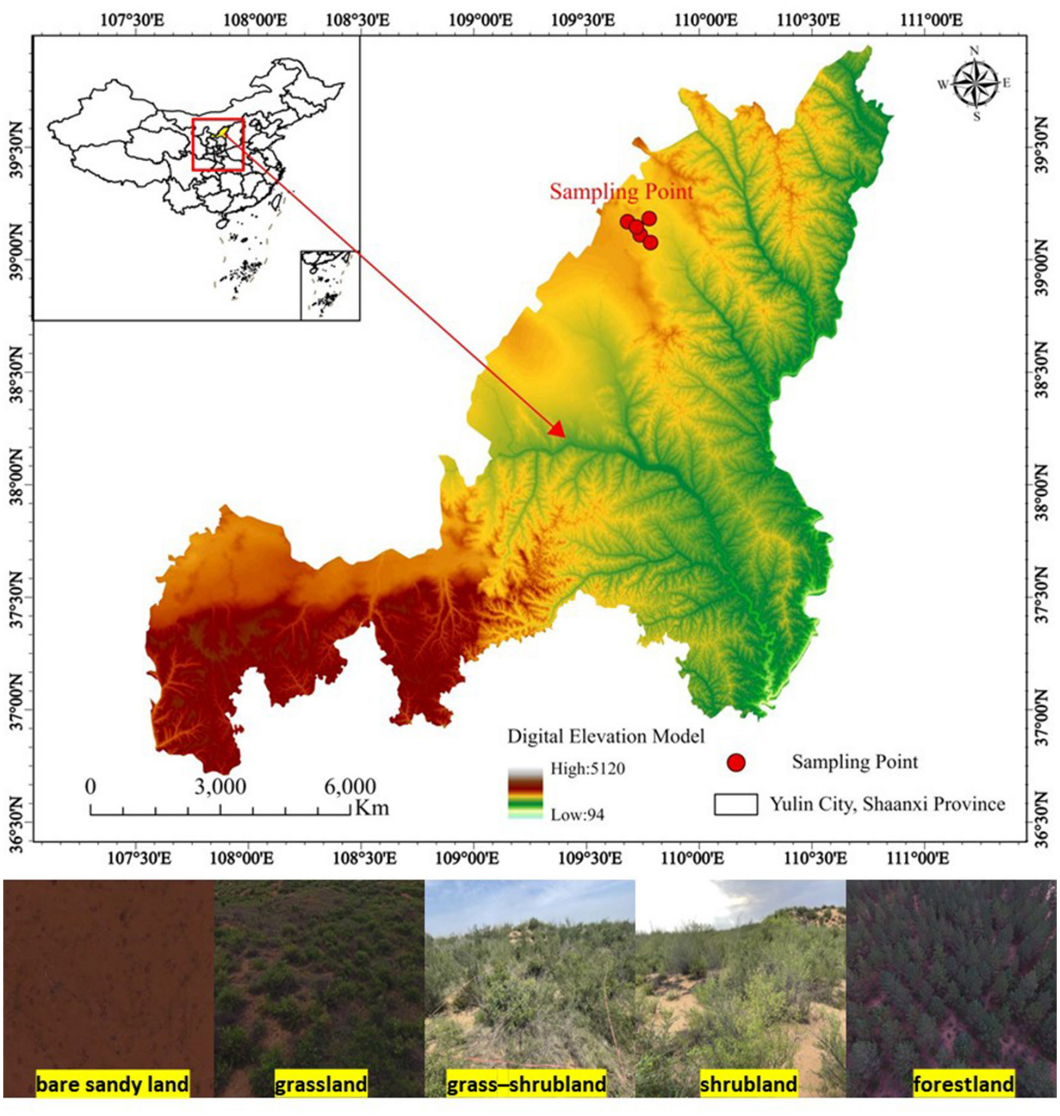
Location map of study area.

The study was conducted at the afforestation base of the Shenmu Ecological Association in the southeastern Mu Us Sandy Land, Shenmu City, Shaanxi Province, China (109°22′E, 38°53′N, Figure 1), at an elevation of 1,100–1,300 m. The region has a temperate semi-arid continental monsoon climate, with a mean annual temperature of approximately 10°C (Chen et al., 2023). The soil is aeolian sandy soil, characterized by a loose texture, low fertility, and poor water- and nutrient-holding capacity. According to the Chinese Soil Taxonomy (CST) and the FAO/WRB system, it is classified as Arenosols and is dominated by sand with very low clay and silt contents. The present landscape is dominated by plantations and secondary successional vegetation.

Field sampling was carried out in July 2022 across five vegetation restoration types representing a restoration gradient: bare sandy land (BSL), grassland (GL), grass–shrubland (GSL), shrubland (SL), and forestland (FL). Plot sizes were set according to vegetation structure and plant density, with 3 m × 3 m plots for BSL and GL, 5 m × 5 m plots for GSL and SL, and 10 m × 10 m plots for FL. Three replicate plots were established for each vegetation type, with approximately 20 m between adjacent plots. After removing surface litter, dead leaves, and stones, soil samples were collected from the 0 cm to 20 cm layer using a sterilized spiral auger following an S-shaped sampling pattern. At each sampling point, six soil cores were collected and thoroughly homogenized to form one composite sample, and three independent composite samples were obtained from each plot.

Each composite sample was divided into three subsamples. One subsample was air-dried and sieved through a 2 mm mesh for soil physicochemical analyses and sulfur fraction measurements (TS, ${\text{SO}}_{4}^{2}$-S, and OS). A second subsample was stored at 4°C for soil microbial biomass determination. The third subsample was frozen at −80°C and transported to the laboratory for DNA extraction and metagenomic analyses related to sulfur cycling.

### Soil physicochemical properties and sulfur pool measurements

Fresh soil samples were used to determine soil water content (SWC). Briefly, subsamples were oven-dried at 105°C for 24 h to constant weight, and SWC was calculated from the mass loss during drying. Air-dried soils passed through a 2 mm sieve were used for soil physicochemical and sulfur pool analyses. Soil pH was measured using a pH meter in a 1:2.5 (w/v) soil-to-water suspension, and electrical conductivity (EC) was determined using a conductivity meter. Soil organic carbon (SOC) was measured by the potassium dichromate external heating method, and total nitrogen (TN) was determined by the Kjeldahl method. All measurements were performed with blanks and replicate samples for quality control. To characterize soil sulfur pools, total sulfur (TS), sulfate sulfur (${\text{SO}}_{4}^{2}$-S), and organic sulfur (OS) were determined. TS was measured using an elemental analyzer combustion method. ${\text{SO}}_{4}^{2}$-S was extracted with a calcium phosphate solution and determined by the BaSO4 turbidimetric method. OS was calculated by difference as OS = TS – ${\text{SO}}_{4}^{2}$-S.

### Microbial biomass and enzyme activity

Fresh soils stored at 4°C were used for microbial biomass and enzyme assays. Soil microbial biomass carbon (MBC) was determined using the chloroform fumigation–extraction approach. Briefly, fumigated and non-fumigated subsamples were extracted with 0.5 mol·L^−1^ K_2_SO4, and organic carbon in the extracts was quantified using a TOC analyzer (TOC-VCHP). Microbial biomass carbon was calculated from the difference between fumigated and non-fumigated extracts and expressed as mg·kg^−1^ (dry soil basis). Soil sucrase activity (SUA) was determined using the 3,5-dinitrosalicylic acid (DNS) colorimetric method, and expressed as mg·g^−1^·24 h^−1^ (dry soil basis).

### DNA extraction, metagenomic sequencing, and assembly

Total genomic DNA was extracted from approximately 0.5 g of soil stored at −80°C using a commercial soil DNA extraction kit according to the manufacturer's instructions. DNA concentration and purity were assessed using a NanoDrop 2000 spectrophotometer based on the A260/280 and A260/230 ratios, and DNA integrity was further verified by 1% agarose gel electrophoresis. Only DNA samples showing intact bands and A260/280 ratios between 1.8 and 2.0 were used for library preparation. High-quality DNA was sheared to an average fragment size of approximately 350 bp, followed by end repair, A-tailing, adapter ligation, and purification. Libraries were amplified using a limited number of PCR cycles, and their size distribution and integrity were evaluated using an Agilent 2100 Bioanalyzer. Library concentrations were quantified with a Qubit fluorometer, and qualified libraries were sequenced on the Illumina NovaSeq 6000 platform in paired-end mode to generate raw metagenomic data for each sample.

Raw paired-end reads were quality-filtered and adapter-trimmed using fastp v0.24.0 (Chen et al., 2018). The resulting clean reads were assembled individually for each sample using MEGAHIT v1.2.9 (Li et al., 2015) with the parameter –min-contig-len 500. Open reading frames (ORFs) were predicted from assembled contigs using Prodigal v2.6.3 (Hyatt et al., 2010) in metagenomic mode. The predicted genes were then dereplicated using CD-HIT v4.8.1 (Fu et al., 2012) at 95% sequence identity and 90% coverage to construct a non-redundant gene catalog, and representative sequences from each cluster were retained for downstream analyses.

### Identification and taxonomic annotation of sulfur-cycling genes

To identify sulfur-cycling genes, unigene protein sequences in the non-redundant gene catalog were annotated against the SCycDB database (Yu et al., 2021) using an e-value threshold of 1 × 10^5^, and only the best hit for each sequence was retained. To estimate the abundance of sulfur-cycling genes, clean reads were mapped back to the non-redundant gene catalog using Salmon (Patro et al., 2017), and gene abundances were normalized as transcripts per million (TPM). Sulfur-cycling genes were classified into nine functional categories based on the SCycDB annotation framework. To characterize the phylum-level taxonomic composition of dominant sulfur-cycling genes, 20 genes were selected according to their total abundance (TPM) across all samples, while ensuring that at least one representative gene was retained from each detected sulfur functional category. This approach allowed the selected genes to reflect both abundance dominance and functional representativeness in the dataset.

To determine the taxonomic affiliations of sulfur-cycling genes, the corresponding protein sequences were searched against the GTDB database (Parks et al., 2022), and taxonomic assignments were generated using the lowest common ancestor (LCA) algorithm. The initial annotations were further integrated with the GTDB bac120 taxonomy reference to recover complete taxonomic lineages from domain to species.

### MAG binning, annotation, and identification of sulfur-related MAGs

To identify potential host microorganisms of sulfur-cycling genes, assembled contigs were subjected to metagenomic binning. Contigs ≥ 1,000 bp were retained and binned using the binning module in MetaWRAP v1.3.2 (Uritskiy et al., 2018). The initial bins were further refined using the bin_refinement module, and only bins with completeness ≥ 50% and contamination ≤ 10% were retained. Bins recovered from all samples were then pooled and dereplicated using dRep v3.5.0 (Olm et al., 2017) at 95% average nucleotide identity (ANI) to obtain a non-redundant set of metagenome-assembled genomes (MAGs). Taxonomic affiliations of MAGs were assigned using the classify_wf workflow in GTDB-Tk v2.4.0 (Parks et al., 2018), and their phylogenetic relationships were inferred using the infer module in GTDB-Tk.

ORFs were predicted from the dereplicated MAG sequences using Prodigal v2.6.3 (Hyatt et al., 2010) in metagenomic mode. The predicted protein sequences were then searched against SCycDB using DIAMOND BLASTp (Buchfink et al., 2021) with an *e*-value threshold of 1 × 10^5^, and only the top hit for each ORF was retained. The types and numbers of annotated sulfur-cycling genes in each MAG were subsequently summarized. MAGs carrying key sulfur-cycling genes were defined as sulfur-related MAGs for downstream analyses.

### Statistical analysis

All statistical analyses were performed in R. Alpha diversity metrics of the microbial community, including Richness and Shannon diversity, were calculated using the vegan package. Mantel tests were performed to assess the relationships between sulfur-cycling functional gene profiles and three groups of environmental variables, including physicochemical properties, sulfur pools and fractions, and microbial diversity, based on pairwise distance matrices.

## Results

### Restoration-driven shifts in soil physicochemical context and sulfur pool partitioning

**Figure 2:**
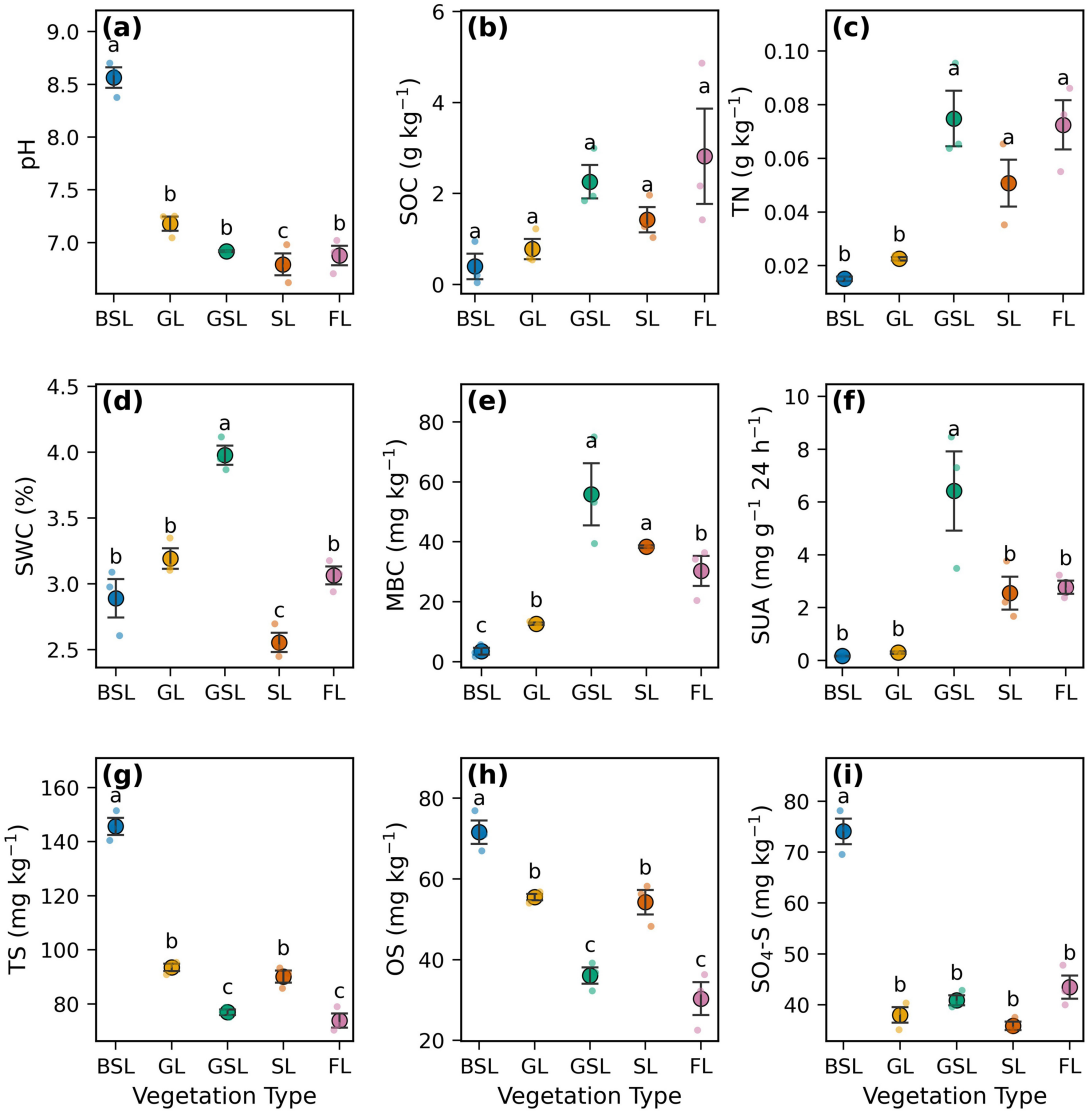
Variation in soil properties and sulfur (S) fractions across different vegetation types. The figure shows changes in soil pH, **(a)** soil organic carbon (SOC), **(b)** total nitrogen (TN), **(c)** soil water content (SWC), **(d)** microbial biomass carbon (MBC), **(e)** sucrase activity (SUA) **(f)** total sulfur (TS), **(g)** organic sulfur (OS), **(h)** and sulfate sulfur (${\text{SO}}_{4}^{2}$-S), **(i)** across five vegetation types: BSL, GL, GSL, SL, and FL. Different letters above the symbols indicate significant differences among vegetation types based on Tukey's HSD test at *P* < 0.05. Error bars represent the standard error (SE).

The five vegetation types, ranging from BSL to FL, represented a clear vegetation restoration gradient and exhibited pronounced differences in soil physicochemical properties and sulfur pool composition (Figure 2). Overall, vegetation restoration significantly reduced soil pH and enhanced nutrient accumulation and microbial activity; however, soil sulfur pools did not increase accordingly, but instead showed clear redistribution patterns.

Regarding soil physicochemical properties, pH was highest in BSL, whereas all restored vegetation types exhibited significantly lower pH values, with the lowest value observed in SL, indicating that vegetation restoration generally alleviated the alkaline characteristics of sandy soils. SOC showed an overall increasing trend along the restoration gradient, although the differences among vegetation types were not statistically significant. In contrast, TN responded more strongly to restoration, with significantly higher values in GSL, SL, and FL than in BSL and GL, suggesting that vegetation restoration promoted nitrogen accumulation and improved the overall soil fertility status.

Soil moisture and microbial activity further revealed clear differences among restoration types in their capacity to regulate the soil microenvironment. SWC was highest in GSL and significantly exceeded that of the other vegetation types, indicating that grass-shrub vegetation was more effective in improving soil water retention. Both MBC and SUA also reached their highest values in GSL and were significantly higher than those in the other vegetation types, suggesting that this mixed vegetation type likely provided more favorable carbon inputs and microenvironmental conditions for microbial growth and enzymatic activity, thereby supporting more active belowground biological processes.

In contrast to the recovery patterns of soil physicochemical properties and microbial activity, soil sulfur pools showed pronounced but non-synchronous responses to vegetation restoration. TS, OS, and ${\text{SO}}_{4}^{2}$-S were all highest in BSL, indicating that sulfur occurred at relatively high total levels in bare sandy soils. With progressing vegetation restoration, both TS and OS declined significantly, with the lowest values observed in FL. By comparison, ${\text{SO}}_{4}^{2}$-S was significantly lower in all restored vegetation types than in BSL, but varied only slightly among the restored vegetation types and remained relatively stable overall. The highly consistent patterns of TS and OS indicate that the restoration-associated decline in total sulfur was driven mainly by depletion of the organic sulfur pool rather than by a parallel decline in sulfate sulfur.

These results suggest that the effect of vegetation restoration on soil sulfur cycling is not reflected by a simple increase in sulfur stocks, but rather by a redistribution of sulfur between organic storage and plant-available pools. This pattern was particularly evident in FL and GSL, where OS declined to levels comparable to or lower than ${\text{SO}}_{4}^{2}$-S, suggesting a greater relative contribution of sulfate to the remaining sulfur pool under restoration. Together with the observed decrease in pH and increases in TN, MBC, and SUA, these results imply that vegetation restoration may stimulate organic sulfur turnover and biological uptake by enhancing rhizosphere inputs and microbial activity. Meanwhile, in the coarse-textured sandy soils of the Mu Us Sandy Land, which are characterized by weak nutrient retention and relatively high sulfate mobility, such changes may contribute to constrained net sulfur accumulation, potentially in combination with sulfate leaching. A similar pattern was observed in GSL, which simultaneously showed the highest SWC, MBC, and SUA but comparatively low TS and OS, consistent with faster sulfur turnover without a corresponding increase in sulfur storage.

Overall, vegetation restoration first reshaped the soil physicochemical and microbial environment, and subsequently altered sulfur partitioning between organic storage and sulfate pools. This asynchrony between general fertility recovery and sulfur pool reorganization indicates that TS alone cannot adequately capture shifts in soil sulfur availability during restoration, and it provides an important environmental basis for interpreting subsequent changes in sulfur-cycling functional genes and key microbial groups.

### Variation in sulfur-cycling functional genes along the vegetation restoration gradient

**Figure 3:**
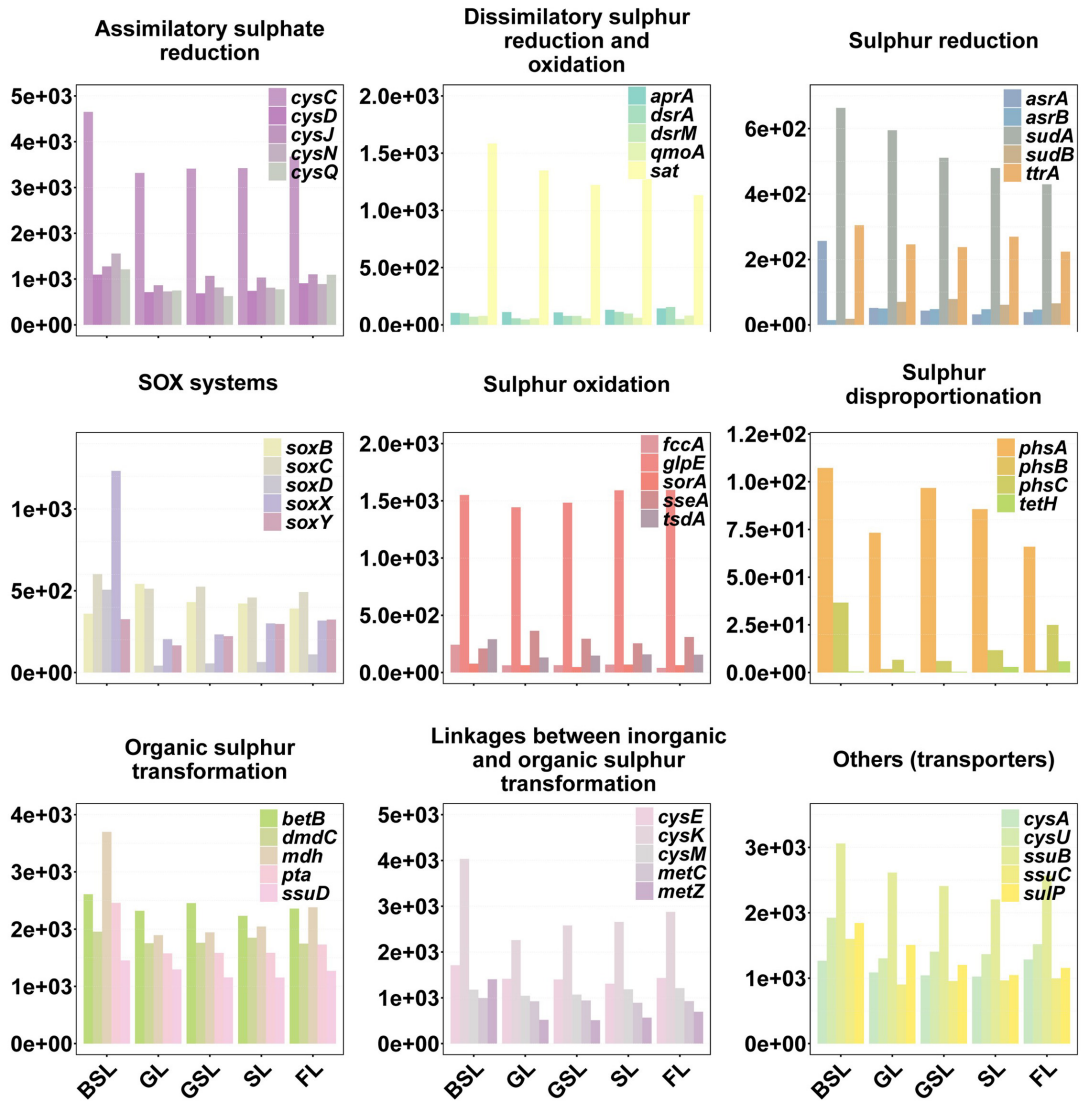
Abundance patterns of dominant sulfur-cycling functional genes across five vegetation types. The figure shows the mean abundance (TPM) of sulfur-cycling functional genes across five vegetation types (BSL, GL, GSL, SL, and FL). A total of nine sulfur-cycling functional categories are included, and only the five most abundant genes within each category were retained for visualization, except for sulfur disproportionation, which contained only four dominant genes. Different colors denote different genes within each functional category, and bar heights represent the mean values of three biological replicates for each vegetation type.

Figure 3 shows that the abundance of sulfur-cycling functional genes did not increase synchronously with vegetation restoration, but instead was generally higher in BSL and lower in the restored vegetation types, although clear differences existed among functional processes. Across the nine sulfur-cycling functional categories, this overall pattern was broadly consistent: most categories showed relatively high abundance in BSL, but generally declined in GL, GSL, and SL. In contrast, some functional categories and dominant genes showed varying degrees of recovery in FL, although no continuous increase along the restoration gradient was observed. These results indicate that changes in soil sulfur-cycling functional genes during vegetation restoration were mainly characterized by a reorganization of functional processes and dominant gene composition, rather than by an overall synchronous increase.

At the functional-category level, assimilatory sulfate reduction and the linkages between inorganic and organic sulfur transformation showed comparatively high abundance and similar variation patterns among vegetation types. Within assimilatory sulfate reduction, *cysC, cysN, cysQ*, and *cysJ* all remained relatively abundant in BSL, with *cysC* being the most prominent. Likewise, *cysE, cysK*, and *metZ*, involved in the linkages between inorganic and organic sulfur transformation, also showed relatively high abundance in BSL, with *cysK* as the dominant gene. These results suggest that genes related to sulfate activation, reduction, and the synthesis of sulfur-containing organic compounds were generally more abundant in bare sandy land, declined in the restored vegetation types, and partially increased again in FL.

A similar pattern was observed for functional categories associated with inorganic sulfur redox transformation, including dissimilatory sulfur reduction and oxidation, s sulfur reduction, and the SOX system, all of which showed overall higher abundance in BSL. In the dissimilatory sulfur reduction and oxidation category, *sat* was the dominant gene and reached its highest abundance in BSL. In the sulfur reduction category, *sudA* and *ttrA* were the major dominant genes, both peaking in BSL, while *ttrA* showed a relatively smaller decline in the restored vegetation types. In the SOX system, *soxX* had the highest abundance and was clearly more abundant in BSL than in the other vegetation types. Overall, these genes involved in inorganic sulfur transformation were generally less abundant in the restored vegetation types than in BSL, indicating a higher inferred functional potential for inorganic sulfur redox transformation in bare sandy land, rather than direct evidence of higher in situ activity.

In addition, genes related to organic sulfur transformation and transporter-associated processes also showed relatively high abundance across vegetation types, but their variation patterns were more stage-dependent. In the organic sulfur transformation category, *betB* and *mdh* were the dominant genes. Among them, *mdh* decreased in the restored vegetation types but increased again in FL, whereas *betB* showed relatively limited variation. Among the transporter-related genes, *ssuB* consistently showed the highest abundance and exhibited a similar pattern of decline followed by recovery. This suggests that genes involved in organic sulfur utilization and transport were more abundant in bare sandy land, but showed a certain degree of functional recovery in the later restoration stage.

When considered together with Figure 2, the variation in sulfur-cycling functional genes was more closely aligned with changes in soil sulfur pool composition. Along vegetation restoration, soil pH decreased, whereas TN, MBC, and SUA increased; in contrast, TS and OS showed overall declines, while ${\text{SO}}_{4}^{2}$-S varied only slightly. Correspondingly, most sulfur-cycling functional genes in Figure 3 reached relatively high abundance in BSL but declined in the restored vegetation types. Notably, although GSL exhibited relatively high MBC, and SUA, it did not show higher abundance of sulfur-cycling genes; several dominant genes, including *cysC, sat*, and *soxX*, were all lower in GSL than in BSL. Overall, the composition of soil sulfur-cycling functional genes differed markedly among vegetation types, with BSL showing higher abundance of sulfur-cycling functional genes.

### Phylum-level taxonomic composition and MAG-based host affiliation of dominant sulfur-cycling genes

**Figure 4:**
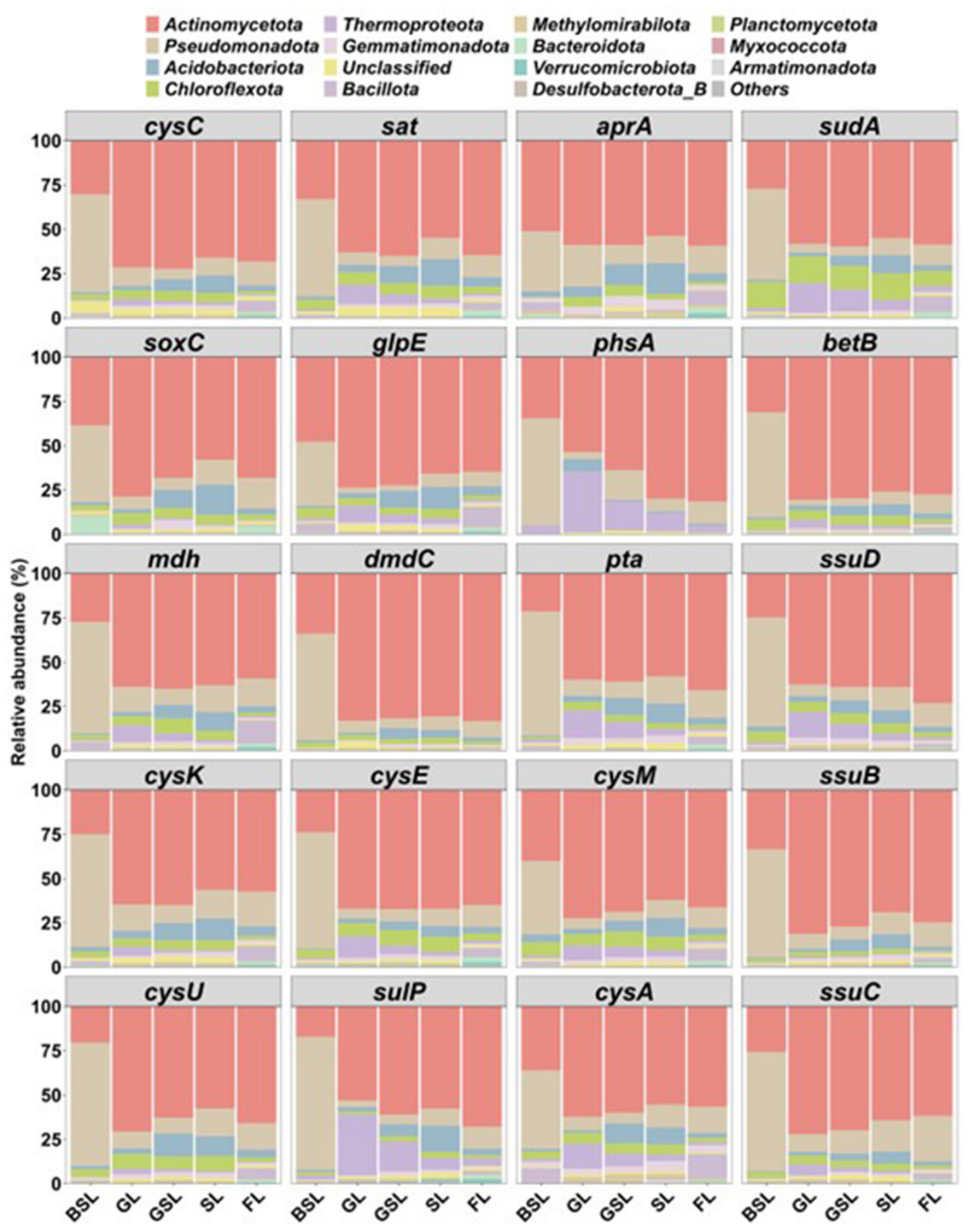
Phylum-level taxonomic composition of dominant sulfur-cycling genes across five vegetation types. The figure shows the phylum-level relative composition of 20 dominant sulfur-cycling genes across five vegetation types (BSL, GL, GSL, SL, and FL). The displayed genes were selected based on their total abundance (TPM) across all samples, while ensuring that at least one representative gene was retained from each detected sulfur functional category. For each gene, the mean TPM across three biological replicates was first calculated for each vegetation type, and the phylum-level contributions were then normalized to 100%. Different colors represent different phyla. For clarity, the 15 most abundant phyla were retained as separate categories, whereas all remaining phyla were grouped as Others.

Figure 4 shows that the 20 dominant sulfur-cycling genes exhibited a broadly conserved yet vegetation-type-dependent phylum-level composition across the five vegetation types. Overall, *Actinomycetota* was the major affiliated phylum for these dominant genes and consistently represented the largest fraction across vegetation types, whereas *Pseudomonadota* generally constituted the second largest component and was particularly prominent in BSL. For several representative genes, including *cysC, sat, mdh, cysK*, and *sulP*, BSL typically displayed a relatively simple taxonomic structure dominated by *Actinomycetota* together with a substantial *Pseudomonadota* contribution. In contrast, the restored vegetation types showed more partitioned phylum-level compositions, with increased contributions from *Acidobacteriota, Chloroflexota*, and, for some genes, *Thermoproteota*, indicating a clear reorganization of the host taxonomic structure of dominant sulfur-cycling genes along vegetation restoration.

At the functional-category level, genes involved in sulfate activation, assimilatory reduction, and linkages between inorganic and organic sulfur transformation remained predominantly associated with *Actinomycetota*, as exemplified by *cysC, cysE, cysK*, and *cysM*, whereas the restored vegetation types generally showed higher relative contributions from *Acidobacteriota* and *Chloroflexota* than BSL. Genes related to inorganic sulfur redox processes showed stronger taxonomic differentiation among vegetation types; for example, *sat, aprA, sudA, soxC*, and *glpE* generally exhibited a more even multi-phyla distribution in the restored communities. Among these genes, *phsA* displayed the most pronounced shift, with a markedly increased contribution from *Thermoproteota* in GL and some restored vegetation types, whereas *Actinomycetota* remained dominant in BSL and FL. By comparison, genes associated with organic sulfur transformation and sulfur transport, such as *pta, ssuD*, and *sulP*, were also mainly affiliated with *Actinomycetota*, but their secondary host groups varied substantially among vegetation types, indicating stronger vegetation-type dependence in host composition. In contrast, genes such as *betB* and *dmdC* retained relatively stable *Actinomycetota*-dominated profiles across vegetation types. The remaining phyla generally accounted for minor proportions and were largely gene-specific.

**Figure 5:**
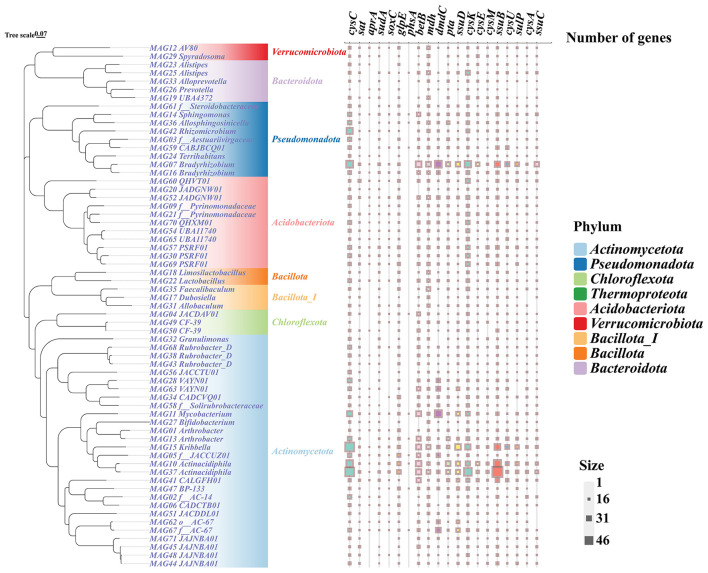
Phylogenetic distribution of sulfur-cycling genes across metagenome-assembled genomes (MAGs). The **(Left)** panel shows the phylogenetic relationships among MAGs carrying dominant sulfur-cycling genes at the genus level, with adjacent taxonomic annotations indicating their phylum-level affiliations. The **(Right)** panel shows the distribution of 20 dominant sulfur-cycling genes across individual MAGs. Square size indicates the number of corresponding genes detected in each MAG, and colors denote the phylum to which each MAG belongs. Only MAGs containing at least one dominant sulfur-cycling gene are shown.

The MAG-based analysis further supported these phylum-level patterns (Figure 5). MAGs carrying sulfur-cycling genes were distributed across multiple phyla, but were concentrated mainly in *Actinomycetota, Pseudomonadota*, and *Acidobacteriota*. At finer taxonomic resolution, the corresponding hosts were mainly affiliated with actinobacterial lineages such as *Arthrobacter, Kribbella*, and *Mycobacterium*, proteobacterial lineages including *Sphingomonas, Bradyrhizobium*, and *Rhizomicrobium*. In terms of gene distribution, *cysC, cysE, cysK, cysM, ssuD, cysU, sulP*, and *pta* were detected across a broader range of MAG hosts, whereas *aprA, sudA*, and *phsA* were restricted to a narrower set of host lineages. Overall, the MAG-based host assignments were consistent with the phylum-level composition shown in Figure 4 and further indicated that dominant sulfur-cycling genes were phylogenetically widespread but unevenly distributed among specific host taxa.

### Co-variation of sulfur-cycling genes and their environmental associations

**Figure 6:**
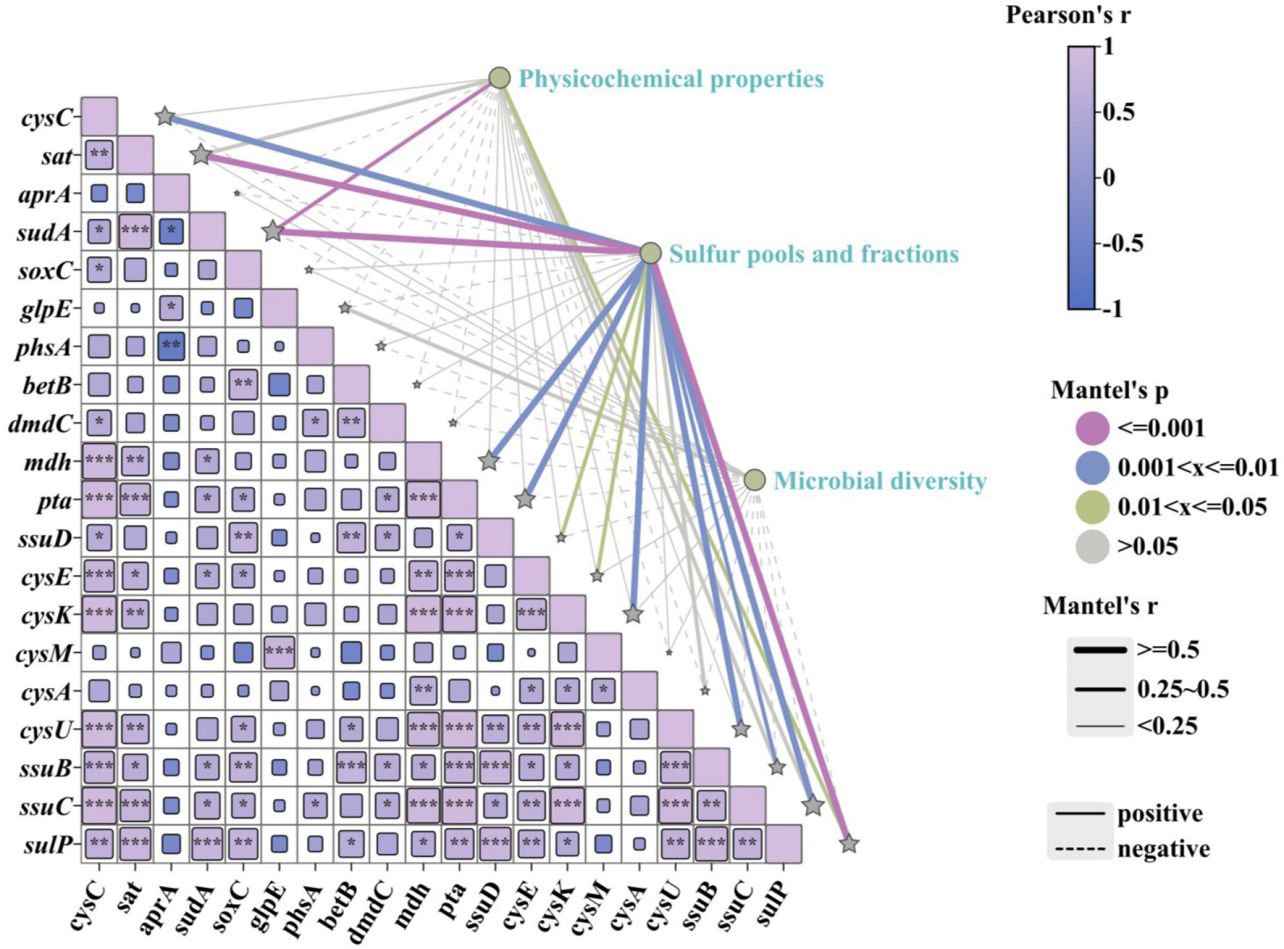
Associations of sulfur-cycling functional genes with soil environmental variables and inter-gene co-variation patterns. The lower triangular matrix shows pairwise Pearson correlations among dominant sulfur-cycling functional genes, with square color indicating the direction and strength of the correlation coefficient (Pearson's *r*). Asterisks indicate significant correlations. The network on the right summarizes Mantel test results linking sulfur-cycling genes to three groups of environmental variables, including physicochemical properties, sulfur pools and fractions, and microbial diversity. Edge color indicates Mantel's *p*-value, edge width represents Mantel's *r*, and solid vs. dashed lines denote positive and negative associations, respectively.

Pearson correlation analysis combined with Mantel tests revealed pronounced coordinated variation among dominant sulfur-cycling functional genes and their close associations with soil sulfur fractions, physicochemical properties, and microbial diversity (Figure 6). Overall, dominant sulfur-cycling genes were not independently distributed, but instead exhibited strong co-variation patterns, indicating widespread functional coupling among different sulfur transformation processes.

At the gene level, the correlation matrix showed that significant positive correlations predominated among most dominant sulfur-cycling genes, with the strongest coordination occurring among genes involved in the linkages between inorganic and organic sulfur transformation and sulfur transport. For example, *cysE, cysK, cysU, ssuB*, s*suC*, and *sulP* formed a tightly connected positive-correlation structure, and these genes were also strongly correlated with several organic sulfur transformation genes, such as *pta* and *ssuD*. These patterns indicate that genes related to sulfate activation, sulfur transport, organic sulfur utilization, and inorganic-organic sulfur linkages tended to vary in a concerted manner, implying that these functional processes may respond jointly to environmental change as coordinated modules in soil. In contrast, genes associated with some dissimilatory sulfur transformation processes, such as *aprA, sudA*, and *phsA*, showed comparatively weaker relationships with other genes, together with some negative or more selective associations, suggesting that they may be subject to stronger ecological constraints.

Mantel tests further showed that, among the three groups of environmental variables, soil sulfur pools and fractions showed the strongest and most widespread associations with dominant sulfur-cycling genes. Compared with physicochemical properties and microbial diversity, sulfur pools and fractions were linked to a larger number of genes by stronger and more significant associations, suggesting that variation in sulfur quantity and composition was more closely linked to dominant sulfur-cycling gene patterns. In particular, several abundant genes, including *cysC, sat, sudA, mdh, pta, cysK, cysU, ssuB, ssuC*, and *sulP*, showed strong associations with sulfur pools and fractions, indicating that variation in total sulfur, organic sulfur, and sulfate was closely associated with the compositional pattern of dominant sulfur-cycling genes. By comparison, physicochemical properties were also significantly associated with part of the gene set, but both the number and strength of these links were generally weaker than those of sulfur fractions. Microbial diversity, in contrast, was associated with only a limited subset of genes, suggesting a weaker overall association with dominant sulfur-cycling gene structure.

Taken together, these results indicate that dominant sulfur-cycling functional genes formed a clear co-variation network in soil, and that their variation was unlikely to be random, but was more likely driven by common environmental filtering. In particular, soil sulfur pools and fractions played a dominant role in structuring sulfur-cycling gene composition, whereas physicochemical properties and microbial diversity provided supplementary regulation. These findings further suggest that, during vegetation restoration, the response of sulfur-cycling genes was reflected not only in changes in the abundance of individual genes, but also in the reorganization of gene network structure and its coupling with the soil sulfur environment.

## Discussion

Vegetation restoration in the Mu Us Sandy Land did not lead to a simple increase in soil sulfur stocks, but instead drove a pronounced reallocation between organic sulfur and sulfate pools. In our study, restored vegetation types showed lower pH and higher TN, MBC, and SUA, indicating clear recovery of the soil physicochemical and microbial context; however, TS and OS declined overall, whereas ${\text{SO}}_{4}^{2}$-S changed much less strongly. This reveals a clear decoupling between general soil fertility recovery and sulfur pool dynamics. Such a pattern is biogeochemically plausible because sulfur in soils is stored predominantly in organic forms, whereas sulfate is the more mobile and directly bioavailable inorganic fraction, and the transformation between these pools is continuously regulated by microbial mineralization, immobilization, and redox processes (Chaudhary et al., 2023; Kertesz and Mirleau, 2004; Wilhelm Scherer, 2009). Recent work has further shown that dissolved or labile organic sulfur is not merely a passive storage pool, but can be rapidly decomposed into sulfate by microorganisms and thereby enter plant–microbe competition and internal soil sulfur cycling (Ma et al., 2024). These results therefore indicate that, in dryland restoration, sulfur recovery should be evaluated from a pool-resolved perspective that distinguishes sulfur storage from sulfur availability, rather than from total sulfur alone (Kimmell et al., 2023; Young et al., 2021).

These shifts in sulfur pools were accompanied by clear restructuring of sulfur-cycling functional potential and host composition. Most dominant sulfur-cycling genes were more abundant in BSL, declined across restored vegetation types, and showed only partial recovery in FL, indicating selective reorganization of sulfur transformation pathways rather than a uniform strengthening of sulfur-cycling potential. Recent syntheses emphasize that microbial sulfur metabolism is inherently process-specific, with assimilatory sulfate reduction, dissimilatory sulfur reduction, sulfur oxidation, and organic sulfur acquisition differing substantially in ecological niche, substrate dependence, and host distribution (Zhou et al., 2025). In this study, dominant genes remained mainly affiliated with *Pseudomonadota*, whereas restored vegetation types showed greater contributions from *Acidobacteriota*, suggesting host redistribution during vegetation restoration. This pattern was further supported by MAG-based host distributions: genes involved in sulfate activation, sulfur transport, and organic sulfur utilization occurred across a broader range of hosts, whereas *aprA, sudA*, and *phsA* were restricted to fewer lineages, indicating stronger ecological specialization of some dissimilatory sulfur pathways. This pattern is consistent with previous studies showing marked ecological differentiation among sulfur-cycling processes and microbial groups (Friedrich et al., 2005; Muyzer and Stams, 2008; Zhou et al., 2025). It should also be noted that the metagenomic profiles presented here reflect genomic potential rather than direct measurements of transcriptional expression or process activity; accordingly, the observed differences among vegetation types should be interpreted as shifts in inferred functional potential rather than confirmed differences in in situ metabolic activity.

Dominant sulfur-cycling genes also showed clear co-variation patterns, and these patterns were more strongly associated with sulfur pools and fractions than with broader physicochemical properties or microbial diversity. Pearson and Mantel analyses showed tight positive correlations among genes such as *cysE, cysK, cysU, ssuB, ssuC*, and *sulP*, which were further linked to organic sulfur utilization genes such as *pta* and *ssuD*. This pattern is consistent with previous studies showing that sulfate activation, sulfur transport, and the utilization of sulfate esters and sulfonates usually do not operate in isolation, but instead function as an integrated system tightly coupled to microbial sulfur demand (Kertesz, 2000; Ma et al., 2024; Park et al., 2020). Mantel analysis further showed that sulfur pools and fractions were the strongest environmental correlates of sulfur-cycling gene structure, indicating that the quantity and composition of sulfur resources were more closely linked to sulfur-cycling functional organization than general fertility variables alone. This result suggests that the response of sulfur cycling to vegetation restoration is expressed not only through shifts in individual genes, but also through reorganization of the linkage between sulfur fractions, functional genes, and host taxa.

It should be noted that this study is based on a field restoration gradient sampled at a single time point and therefore follows a space-for-time framework. Although this design is useful for identifying broad ecological patterns, differences among vegetation types may reflect not only restoration stage, but also variation in plant composition, site history, microtopography, and other unmeasured environmental factors. Accordingly, some causal interpretations of restoration effects should be made with caution.

## Conclusion

In conclusion, vegetation restoration in the Mu Us Sandy Land did not simply enhance soil sulfur accumulation, but instead reshaped sulfur allocation and sulfur-cycling functional potential in a stage-dependent manner. Along the restoration gradient, total sulfur and organic sulfur declined, whereas sulfate remained comparatively stable, indicating that sulfur recovery was not synchronized with the general recovery of soil fertility. At the microbial level, dominant sulfur-cycling genes declined in restored vegetation types and showed only partial recovery in forestland, pointing to selective reorganization of sulfur transformation pathways rather than a uniform enhancement of sulfur-cycling potential. Together with the taxonomic, MAG-based, and co-variation results, these findings suggest that sulfur recovery during dryland vegetation restoration is better understood as a process of sulfur pool reallocation, microbial functional reorganization, and host-level ecological redistribution than as simple sulfur accumulation. We therefore propose that a pool-resolved, function-oriented framework integrating sulfur fractions, sulfur-cycling genes, and their host taxa provides a more robust basis for evaluating belowground nutrient recovery in semi-arid restoration ecosystems.

## Data Availability

The data presented in the study are deposited in the NCBI repository, accession number PRJNA1469017.
